# PCR mediated recombination impacts the analysis of hepatitis B Virus covalently closed circular DNA

**DOI:** 10.1186/s12977-016-0318-1

**Published:** 2016-12-20

**Authors:** Rodolphe Suspène, Valérie Thiers, Jean-Pierre Vartanian, Simon Wain-Hobson

**Affiliations:** Molecular Retrovirology Unit, Institut Pasteur, 28 rue du Dr. Roux, 75724 Paris Cedex 15, France

**Keywords:** HBV, cccDNA, rcDNA, PCR recombination

## Abstract

**Background:**

The replication of HBV involves the production of covalently closed circular DNA (cccDNA) from the HBV genome through the repair of virion relaxed circular DNA (rcDNA) in the virion. As cccDNA is the transcription template for HBV genomes, it needs to be eliminated from hepatocytes if the eradication of chronic HBV infection is to be achieved. PCR quantitation of cccDNA copy number is the technique of choice for evaluating the efficiency of treatment regimens. The PCR target commonly used to identify cccDNA spans the gapped region of rcDNA and is considered to accurately distinguish between cccDNA and rcDNA. There is however, a potentially confounding issue in that PCR can generate larger targets from collections of small DNA fragments, a phenomenon known as PCR recombination.

**Results:**

The impact of PCR recombination towards the amplification of this cccDNA specific target was explored by mixing three marked, yet overlapping HBV DNA fragments. Thirteen of sixteen possible recombinants were identified by sequencing with frequencies ranging from 0.6 to 23%. To confirm this finding in vivo, HBV positive sera were treated with DNase I and submitted to quantitative real-time PCR. Under these conditions, it was possible to amplify the cccDNA specific segment without difficulty. As the virion contains uniquely rcDNA, amplification of the cccDNA target resulted from PCR recombination.

**Conclusions:**

PCR quantitation of cccDNA may be more difficult than hitherto thought. Current detection protocols need to be investigated so as to help in the management of chronic HBV infection.

## Findings

Incoming partly double stranded hepatitis B virus (HBV) DNA genomes are completed by the viral polymerase generating relaxed circular DNA (rcDNA). Following translocation to the nucleus, the gaps are repaired by the host repair system to generate covalently closed circular DNA (cccDNA), the template for the HBV pregenome and viral mRNAs. Consequently, catabolism of cccDNA is necessary for the eradication of chronic HBV infection. cccDNA levels are a useful biomarker of HBV replication although its clinical use is restricted as it requires invasive sampling. PCR quantitation of cccDNA is widely used to assess the effectiveness of antiviral therapy or in the analysis of anti-HBV restriction factors [1–4].

As the amount of HBV rcDNA in the cell is much higher than cccDNA, distinguishing the latter is a challenge. The signal differences between the two forms are the two gaps in rcDNA. PCR across this region has been considered to selectively amplify cccDNA. In the later cycles of a PCR reaction, where [DNA] > [Taq], not all strands are completed. This is highlighted by addition of a 10 min step at the end of the reaction. During these later cycles, incomplete DNA strands can switch templates resulting in the formation of recombinant DNA strands, a phenomenon referred to as PCR mediated recombination [5]. This is not detectable when the target is genetically homogenous but is readily seen for HIV where there is considerable intra-sample genetic heterogeneity [6]. Similarly “DNA shuffling” generates large recombinant DNA molecules from smaller DNA fragments through template switching [7]. Here strand switching occurs by the second round of PCR and larger single stranded DNA molecules are built up extending from the outer primers until they overlap. From that point on classical PCR takes over. The amplification HBV cccDNA is akin to DNA shuffling.

We wondered whether PCR mediated recombination during amplification of the double gapped region of HBV rcDNA could generate the target sequence considered to be specific for cccDNA.

**Fig. 1:**
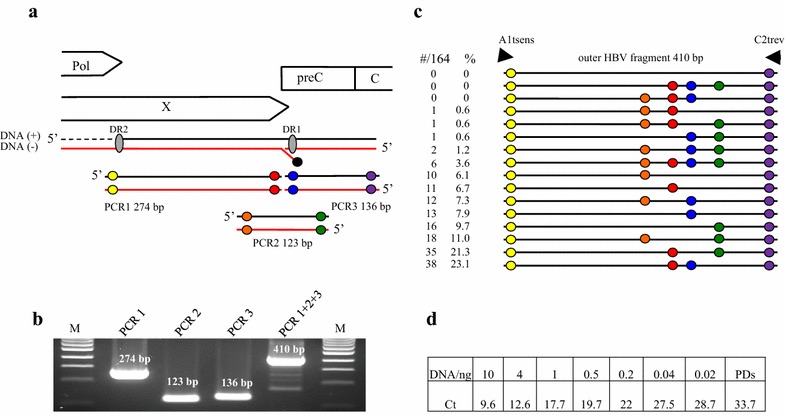
Recombinant forms of HBV in vitro and in vivo. **a** The different open reading frames encoded by the genome, designated as polymerase (Pol), core and X are aligned to the three PCR fragments used during the PCR. The HBV polymerase covalently bound to the (−) DNA strand is shown by a *black circle*. *Coloured circles* represent differences in nucleotides from the input pCayw plasmid (*yellow* TG- > AG and CTG- > GAC, *orange* TT- > AA; *red* AC- > TG, *blue* TT- > AA, *green* GT- > CA, *purple* AG- > TC and TCC- > AGG). **b** PCR1, PCR2 and PCR3 were amplified under standard conditions. PCR1 + 2 + 3 represented the 410 bp recombinant forms. *M* molecular weight markers. **c** Collection of 164 PCR mediated recombinants. The order of the *coloured circles* distinguishes the different recombinant forms. The number and the frequency of recombinants are presented on the *left*. **d** Quantification by real-time PCR based on SYBR Green on serial dilution of the three PCR fragments. All fragments correspond to the 410 bp fragment. Only primer-dimers (PDs) were detected in the absence of DNA

To test this, three different overlapping HBV fragments simulating the double gapped structure of rcDNA were PCR amplified (PCR1 positions 1545–1818; PCR2 positions 1765–1887; PCR3 position 1819–1954; accession number NC_003977.2; Fig. 1a). Primers incorporated mutations (underlined) to distinguish PCR fragments from input HBVayw DNA. Primers were A1sens, 5′GACAGCCCGTCTGTGCCTTCTCATGACCCGGA and A2rev, 5′CATGCAGCTGGTGCGCAGACCAA for PCR1, B1sens 5′GGTCTAAGTACTAGGAGGCTGTA and B2rev 5′AAGGCTGAGCTTGGAGGCTTGAA for PCR2 and C1sens 5′CAACAATTTCACCTCTGCCTAAT and C2rev 5′ACGTCAGTAACTCCACAGTAGCAGGAAATTCT for PCR3. Amplification conditions were identical for the three fragments and corresponded to 5 min at 95 °C then 30 s at 95 °C, 30 s at 58 °C and 2 min at 72 °C for 35 cycles, being completed by a final incubation at 72 °C for 10 min. Reactions were performed in a final volume of 100 µl.

The three PCR fragments were purified from agarose gels (Qiaex II kit, Qiagen, France), quantified by nanodrop spectrophotometer, mixed on an equimolar basis (100 ng final) and subjected to 35 cycles of amplification with the outer primers A1tsens, 5′GACAGCCCGTCTGTGCCTTCTCATGAC and C2trev, 5′ACGTCAGTAACTCCACAGTAGCAGG using the above PCR conditions. Primers A1t and C2t corresponded to primers A1 and C2 and were shortened respectively by 5 and 7 nucleotides 3′ of the primer to avoid amplification of HBVayw plasmid. As can be seen from Fig. 1b, a 410 bp fragment was readily obtained from the mixture of the 123, 136 and 274 bp fragments. The 410 bp outer PCR products were cloned using the TOPO TA cloning kit (Invitrogen) and individual clones sequenced.

As can be seen in Fig. 1c, all of the 164 sequences were recombinants where coloured circles represent the mutations introduced into the primers. Thirteen of the sixteen possible recombinants were found with frequencies ranging from 0.6 to 23%. The marker mutations, identified by colour in Fig. 1a, were present in roughly the same proportions across the 164 sequences consistent with the fact that equimolar proportions of each DNA fragment were used (orange ~30%, red ~56%, blue ~44% and green ~48%). Some PCR mediated recombinants required just two template switches (one circle) while others required six (4 circles, Fig. 1c). The most complex recombinant—four circles—represented 3.6% of the total. Interestingly, no clones with the HBVayw reference sequence were found (no circles, Fig. 1a) meaning that there was no carry over of input HBV DNA during purification of the amplicons.

To determine the sensitivity of the PCR, we performed real-time PCR based on SYBR Green with serial dilutions of an equimolar mix of the three PCR fragments. Amplification conditions corresponded to 10 min at 95 °C then 20 s at 95 °C, 20 s at 55 °C and 2 min at 68 °C for 40 cycles with primers A1tsens and C2trev. Amplification was completed by a melting curve step and all products were analysed by gel electrophoresis. As can be seen from Fig. 1d, 410 bp PCR recombinants could be detected down to 20 pg input DNA. While this is a rather high threshold, PCR recombination involves the diffusion of large molecules many times larger than typical amplification primers. In addition, a minimum of three cycles are necessary to assemble the 410 bp fragment. Hence, a rather elevated detection threshold is to be expected.

**Fig. 2:**
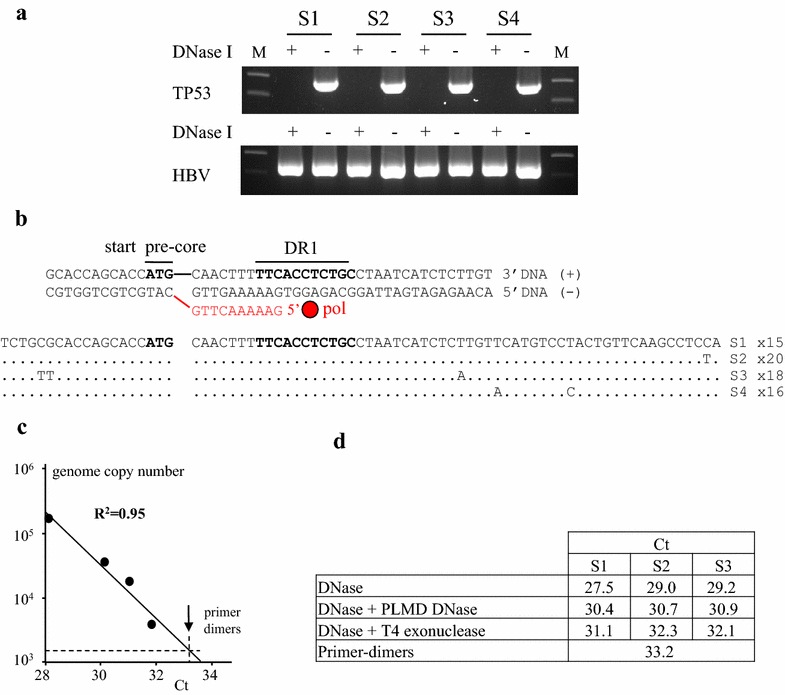
Target cccDNA generated from virion associated rcDNA. **a** PCR analysis of the serum of 4 HBV infected patients (S1–S4) in presence or absence of DNase I treatment. *TP53* amplification (*top*) and HBV (*bottom*). **b** Sequences of HBV from the serum of the 4 infected patients. The sequence from serum 1 was used as reference, the number of sequences analysed is given on the *right*. Above is a representation of rcDNA for this region. **c** Quantification by real-time PCR based on SYBR Green on serum S1. **d** SYBR Green PCR quantification after DNase treatment followed by Plasmid-safe DNase and T4 exonuclease on serum S1, S2 and S3. All amplifications were controlled by gel electrophoresis. Above a Ct of 32.3 only primer-dimers were recovered

In order to demonstrate PCR mediated recombination of *bona fide* HBV rcDNA, sera from 4 HBV infected patients (S1–S4) with high HBV copy numbers (S1 8.42; S2 7.28; S3 8.72, S4 7.53 log UI previously described [8]) were analysed. Serum can harbour circulating genomic DNA and so may contain a little cccDNA from necrotized hepatocytes. To investigate this, sera were split and part was treated with DNase I (5 units, 2 h, 37 °C, heat-inactivated at 95 °C, 10 min, Fig. 2a). *TP53* DNA was targeted using primers P53sens 5′GAGCTGGACCTTAGGCTCCAGAAAGGACA and P53rev 5′GCTGGTGTTGTTGGGCAGTGCTAGGAA, PCR conditions were described [9] while HBV DNA was amplified across the double gap structure using primers HBVsens 5′GACTCCCCGTCTGTGCCTTCTCA and HBVrev 5′ACGAGAGTAACTCCACAGTAGCT. *TP53* DNA could be recovered from untreated, but not DNase I treated sera proving the presence of circulating chromosomal DNA in all four samples. By contrast HBV DNA could be found in both DNase I treated and untreated sera (Fig. 2a). Presumably the HBV signal from the treated sample comes from rcDNA protected by the virion. DNase I treated HBV PCR products were cloned and 15–20 clones sequenced from each sample (Fig. 2b). Sequences were identical within a serum sample but all different between samples proving no cross contamination between samples. As the virion uniquely contains rcDNA, amplification of a 410 bp fragment inevitably required PCR mediated recombination.

To investigate sensitivity, we performed real-time SYBR Green PCR. Serum S1 (viral load of 8.42 log IU/ml) was digested with DNase I, serially diluted and the Ct determined. There was a good linear correlation between log[DNA] and Ct (Fig. 2c) indicating that PCR recombinants could be obtained down to the limits of detection, which is a little lower than 3600 copies. Protocols for quantifying cccDNA usually include a treatment by a Plasmid-Safe DNase. Accordingly, DNase I treated sera S1–S3 were heat inactivated and treated with Plasmid-Safe DNase (3 units, Illumina) or with T4 Exonuclease (3 units, New England BioLabs) for 30 min at 37 °C which is typical of such protocols [10]. Target cccDNA was quantitated by SYBR Green PCR. As can be observed in Fig. 2d, treatment with Plasmid-Safe DNase resulted in a higher Ct value indicating that treatment had eliminated input DNA. T4 exonuclease treatment eliminated even more DNA (Fig. 2d). Despite these treatments, 410 bp target cccDNA could be recovered by PCR recombination, for the input was uniquely rcDNA.

As HBV cccDNA is synonymous with persistent infection, it is both a therapeutic target and an attractive biomarker to follow, while PCR is the obvious technology to employ. Distinguishing rcDNA and cccDNA in the nucleus is extremely difficult. That the former is found in the nucleus is indicated by integrated HBV sequences with integration sites mapping close to the 5′ ends of rcDNA strands [11]. From this point on, PCR mediated recombination becomes an issue for the identification of cccDNA. The use of longer elongation times reduces but does not eliminate PCR recombination [5]. Unambiguous quantitation of cccDNA by PCR may be more difficult than hitherto thought.
